# A Simple Yeast-Based Strategy to Identify Host Cellular Processes Targeted by Bacterial Effector Proteins

**DOI:** 10.1371/journal.pone.0027698

**Published:** 2011-11-15

**Authors:** Eran Bosis, Dor Salomon, Guido Sessa

**Affiliations:** Department of Molecular Biology and Ecology of Plants, George S. Wise Faculty of Life Sciences, Tel Aviv University, Tel Aviv, Israel; Université de Genève, Switzerland

## Abstract

Bacterial effector proteins, which are delivered into the host cell via the type III secretion system, play a key role in the pathogenicity of Gram-negative bacteria by modulating various host cellular processes to the benefit of the pathogen. To identify cellular processes targeted by bacterial effectors, we developed a simple strategy that uses an array of yeast deletion strains fitted into a single 96-well plate. The array is unique in that it was optimized computationally such that despite the small number of deletion strains, it covers the majority of genes in the yeast synthetic lethal interaction network. The deletion strains in the array are screened for hypersensitivity to the expression of a bacterial effector of interest. The hypersensitive deletion strains are then analyzed for their synthetic lethal interactions to identify potential targets of the bacterial effector. We describe the identification, using this approach, of a cellular process targeted by the *Xanthomonas campestris* type III effector XopE2. Interestingly, we discover that XopE2 affects the yeast cell wall and the endoplasmic reticulum stress response. More generally, the use of a single 96-well plate makes the screening process accessible to any laboratory and facilitates the analysis of a large number of bacterial effectors in a short period of time. It therefore provides a promising platform for studying the functions and cellular targets of bacterial effectors and other virulence proteins.

## Introduction

Gram-negative bacteria are the causal agents of numerous diseases in plants and animals. Many of these bacteria encode a syringe-like structure termed the type III secretion system, which delivers effector proteins into the host cell during infection [1]. Once inside the host cell, these virulence proteins, named type III effectors (T3Es), modulate various host cellular processes to the benefit of the pathogen. T3Es were shown to target components of the immune system, transcription, cell death, proteasome and ubiquitination systems, RNA metabolism, hormone pathways and chloroplast and mitochondria functions [2], [3], [4]. A current challenge is to systematically determine the virulence functions, biochemical activities and host targets of T3Es.

The yeast *Saccharomyces cerevisiae* has recently emerged as a tool to investigate bacterial T3Es [5], [6], [7], [8]. The use of yeast in the study of bacterial effectors is based on the observation that these proteins often target fundamental cellular processes that are conserved among all eukaryotes. In agreement with this premise, the expression of many T3Es from plant and animal pathogens inhibits yeast growth [6], [9]. Toxic phenotypes induced by bacterial effectors in yeast were used in suppressor screens for the identification of eukaryotic targets of the effectors [10], [11].

Recently, Kramer et al. described an approach to study bacterial effectors in yeast, which uses yeast synthetic lethal (SL) interaction data [12]. Synthetic lethality is defined as the situation in which two genes that are non-essential when individually mutated cause lethality when they are combined as a double mutant [13]. Kramer et al. systematically screened the yeast deletion strain collection for strains that were hypersensitive to the expression of the *Shigella* T3E OspF, a member of the phosphothreonine lyase family [14]. Their analysis was based on the assumption that phenotypes resulting from the activity of OspF would resemble phenotypes of a mutation in the target gene of the effector. Therefore, there should be an overlap between the deletion strains hypersensitive to the effector and the SL interactions of the target gene. Accordingly, genes were defined as congruent to an effector, if their sets of SL interactions overlapped with the deletion strains hypersensitive to that effector [12], [15]. The congruent genes represent putative targets of the effector. Kramer et al. combined the results from the screen with yeast SL interaction data to identify genes congruent to OspF. Analysis of the processes in which these congruent genes were involved resulted in the identification of a cellular process that was targeted by the effector. Although it can lead to the identification of the cellular targets of T3Es, the major disadvantage of this approach is that it requires the screening of all 4,750 deletion strains, which limits its wide application to laboratories that have the required technology. Alternative methods, such as SLAM (synthetic lethality analysis with microarrays) and diploid-based SLAM, allow for identification of SL interactions in a single pool [16],[17]. However, the use of microarrays increases the complexity of the assay.

In this work, we present a simple strategy that uses yeast SL interaction data to identify cellular processes that are affected by the expression of bacterial T3Es. Our strategy is based on the finding that it is possible to cover the majority of the interacting genes (i.e. genes having at least one known SL interaction) with 90 deletion strains. We show that an array of yeast deletion strains fitted into a single 96-well plate covers 69% of the interacting genes with less than 2% of the deletion strains in the yeast collection. The small number of deletion strains in the array simplifies the analysis, reduces costs and facilitates the screening of a large number of bacterial T3Es in a short period of time.

**Figure 1:**
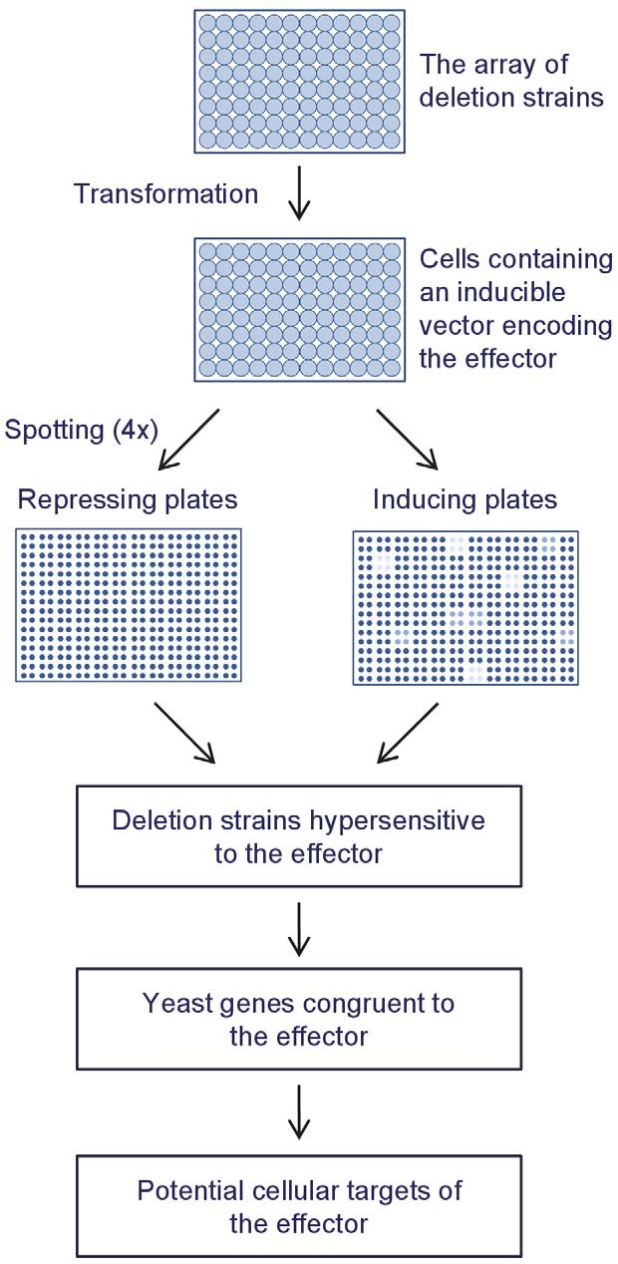
Schematic representation of the approach used in this study. A yeast-based strategy to identify cellular processes targeted by bacterial type III effectors using an array of deletion strains in a single 96-well plate.

The deletion strains are transformed with a galactose inducible expression vector encoding the bacterial T3E of interest and then screened to identify deletion strains that are hypersensitive to the expression of the effector (a schematic representation of our approach is shown in Figure 1). A centromere-containing vector is used to obtain low-level expression of the bacterial T3E and thus to increase the specificity of the assay [18]. The hypersensitive deletion strains are then analyzed to identify genes congruent to the bacterial effector. The pathways and processes enriched among the congruent genes represent potential targets of the bacterial effector. We describe the identification, using this approach, of a yeast cellular process targeted by the *Xanthomonas campestris* pv. *vesicatoria* T3E XopE2. Our approach can be easily employed to characterize T3Es from plant and animal pathogens as well as other virulence proteins that function inside the host cell.

## Results

### Ninety deletion strains are sufficient to cover the majority of the yeast SL interaction network

The yeast SL interaction network contains 10,438 interactions between 2,795 genes based on data extracted from the *Saccharomyces* Genome Database. Our objective was to construct a small array of deletion strains covering the yeast SL interaction network and then use this array to identify cellular processes affected by the expression of bacterial effectors (Fig. 1). Our first goal was to evaluate the minimal number of haploid null deletion strains required for maximal coverage of the yeast SL interaction network. We were interested in two aspects. The first aspect was the number of genes that were covered by the selected deletion strains, and the second aspect was the number of genes that were covered by two or more of the selected deletion strains, to ensure some overlap between the deletion strains. Based on the notion that the distribution of SL interactions between the genes was not equal [13], we hypothesized that maximal coverage of the network should not require the entire collection of deletion strains.

**Figure 2:**
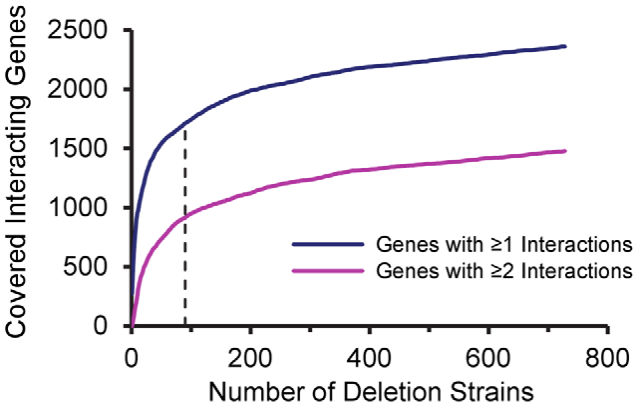
Coverage of the SL interaction network as a function of the number of deletion strains. The selection algorithm iterates over the list of viable deletion strains and every time selects the deletion strain with the maximal contribution to the coverage of the SL network. The selection process stops when the contribution of the next deletion strains is zero. The graph shows the number of interacting genes (i.e. genes having at least one known SL interaction) covered by the deletion strains throughout the selection process. The dashed line marks the coverage by the first 90 deletion strains.

To evaluate the minimal number of deletion strains required for maximal coverage of the SL interaction network, we constructed a selection algorithm that iterated over the list of viable null deletion strains and every time selected the deletion strain that had the maximal contribution to the coverage of the SL interaction network (according to SL interaction data retrieved from the *Saccharomyces* Genome Database). Contribution of a deletion strain was defined as the number of interacting genes (i.e. genes having at least one known SL interaction) that were not covered by the selected deletion strains or covered only once. To increase the efficiency of the algorithm, we discarded deletion strains with no SL interactions as they could not contribute to the coverage of the network. In cases where two or more deletion strains had the same contribution, the algorithm selected one of them randomly. The algorithm continued iterating over the list until the contribution of the next deletion strain was zero, meaning that the selected deletion strains reached maximal coverage of the network (Fig. 2). As expected, the first deletion strains to be selected were those which interacted with the largest number of genes (‘hub genes’). However, with the progress of the selection process, deletion strains which interacted with genes not covered by previously selected deletion strains (or covered once) were favored over deletion strains which interacted with a larger number of genes, but that were already covered. The minimal number of deletion strains required for maximal coverage was found to be 728 out of the 4,750 viable deletion strains (∼15.3%). Altogether, 2,360 interacting genes were covered by the deletion strains, 1,478 of them were covered by two or more deletion strains. Thus, maximal coverage of the yeast SL interaction network required only a small subset of the deletion strains.

Interestingly, our calculations indicated that the increase in interacting genes was not linearly proportional to the number of deletion strains (Fig. 2). In fact, it showed that 90 deletion strains were sufficient to cover 1,712 out of the 2,360 genes (∼72.5%) (dashed line in Fig. 2). This is because the first deletion strains to be selected were hub genes interacting with many genes. Therefore, a single 96-well plate is sufficient to cover the majority of the interacting genes in the yeast SL interaction network.

### Constructing the array of deletion strains

Based on our finding that 90 deletion strains were sufficient to cover the majority of the interacting genes (i.e. genes having at least one known SL interaction) in the yeast SL interaction network, we decided to limit the array of the deletion strains to a single 96-well plate. This decision was driven by the premise that it is much easier to screen a single 96-well plate than to screen over fifty 96-well plates containing the entire yeast deletion strain collection. Our original aim was to select 94 deletion strains, leaving 2 wells for the wild-type strain. We preselected 4 deletion strains of interest (*Δhac1*, *Δire1*, *Δbck1* and *Δslt2*), and the other 90 deletion strains were selected computationally using the selection algorithm described earlier. The algorithm was limited to 90 iterations over the list of viable deletion strains and was devised to take into consideration the contribution of the 4 preselected genes. We corroborated the results using the Genetic Algorithm, an evolution-inspired optimization technique, which we employed in the past to determine the rate constants of chemical-kinetic models [19], [20], [21] (see Text S1). We assessed the ability of the selected deletion strains to grow properly on selective synthetic media containing glucose and on synthetic media containing galactose, which is used in our system to induce the expression of the bacterial effector. Due to poor growth, several deletion strains had to be substituted by other deletion strains covering similar SL interactions or by copies of the wild-type strain (see Text S1). The final 96-well plate contained 92 deletion strains and 4 copies of the wild-type strain (Fig. S1 and Table S1). Remarkably, the final array of the deletion strains, which used less than 2% of the deletion strains in the yeast deletion strain collection, covered 1,624 out of the 2,360 interacting genes in yeast (∼69%) with 833 interacting genes covered by two or more deletion strains.

### The array of deletion strains is sufficient to predict the cellular target of OspF

We noted earlier that Kramer et al. identified the cellular process targeted by the *Shigella* T3E OspF by screening the yeast deletion strain collection for deletion strains hypersensitive to OspF [12]. They showed that almost all of the genes congruent to OspF (*i.e.* genes which have sets of SL interactions overlapping with the deletion strains hypersensitive to the effector) encoded proteins involved in either the cell wall integrity (CWI) pathway or chitin biosynthesis, both of which are processes related to cell wall biogenesis. These findings led to the conclusion that this bacterial effector targeted the CWI pathway. We tested whether our computationally selected array of deletion strains was sufficient to predict the cellular target of OspF, as a proof of concept for our small array approach. Examination of the 83 deletion strains that were determined by Kramer et al. as hypersensitive to OspF revealed that 9 of them were included in our array of deletion strains (*Δccr4*, *Δsmi1*, *Δlas21*, *Δfks1*, *Δgim5*, *Δgas1*, *Δbni1*, *Δkre1* and *Δpop2*). Based on these deletion strains, we identified 13 genes that were congruent to OspF (Table S2; see Text S1 for description of the analysis). Encouragingly, 8 of the 13 congruent genes we identified were also found by Kramer et al. [12]. Moreover, analysis of the Gene Ontology (GO) attributes that were enriched among the 13 congruent genes, which was performed using the FuncAssociate 2.0 web application [22], revealed that the congruent genes were indeed involved in processes related to cell wall biogenesis (see Table S3), suggesting that OspF targeted this cellular process. In conclusion, this result indicates that the array of the deletion strains we constructed can be used to identify processes affected by bacterial effectors, as an efficient alternative to screening the entire yeast deletion strain collection.

### XopE2 is predicted to target cell wall biogenesis and organization

**Figure 3:**
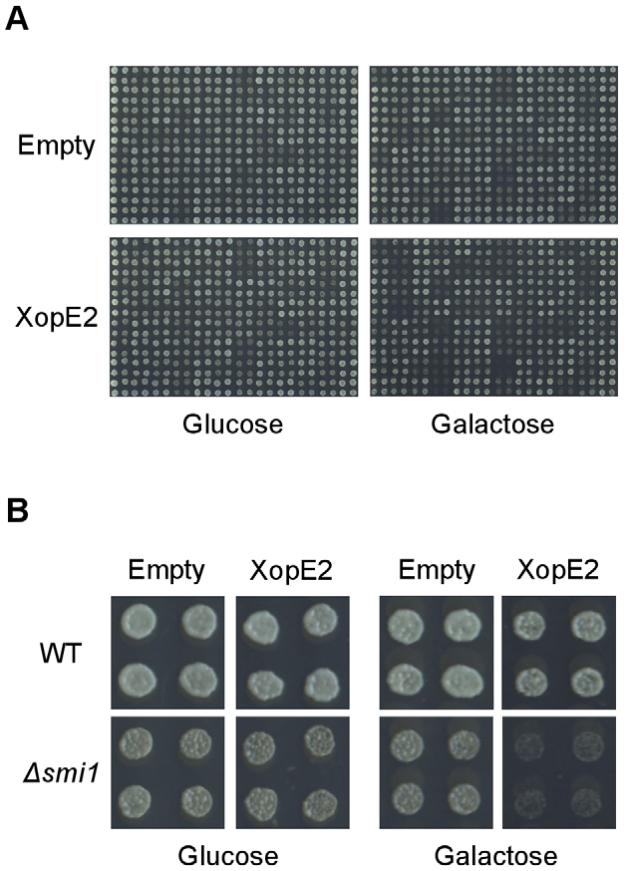
Screen for deletion strains hypersensitive to XopE2. **A,** The deletion strains in the array were transformed with pGML10, either empty or encoding XopE2, and were spotted in quadruplicates on repressing (2% glucose) and inducing (2% galactose and 1% raffinose) media. The plates were scanned after 2–3 days at 30°C to quantify growth. **B,** Quadruplicate spots of the *Δsmi1* deletion strain from the screen described in A.

We next tested whether our approach could be used to predict the cellular targets of bacterial T3Es for which no targets were previously defined. The *Xanthomonas campestris* pv. *vesicatoria* T3E XopE2 is a member of the HopX family of putative transglutaminases that was found to localize to the plasma membrane of plant cells [23]. We used our array of deletion strains to elucidate the cellular processes targeted by XopE2. First, we transformed the array with the yeast galactose inducible expression vector pGML10 [24] either empty or encoding XopE2. We picked the transformed cells into round-bottom microtiter plates containing repressing media and allowed them to grow to saturation. After washing and diluting the saturated cells 1∶10, we spotted the cells on repressing and inducing media and screened them to identify deletion strains that were hypersensitive to the expression of XopE2 (a deletion strain was defined as hypersensitive to XopE2 if the relative growth ratio of the strain was lower than 50% in at least two of the three biological repetitions; see Text S1). Figure 3A shows the various plates from one of three biological repetitions after 2–3 days at 30°C. Figure 3B shows the quadruplicate spots of the *Δsmi1* deletion strain that was identified as hypersensitive to XopE2. Altogether, we identified in the screen 8 hypersensitive deletion strains (*Δslt2*, *Δchs5*, *Δsmi1*, *Δswi4*, *Δcla4*, *Δswf1*, *Δrad27* and *Δnbp2*). The hypersensitivity of these deletion strains to XopE2 was validated using a spotting assay (Fig. S2).

Based on the deletion strains found to be hypersensitive to XopE2, we identified 12 genes that were congruent to XopE2 (*gas1*, *bni1*, *smi1*, *bem2*, *bck1*, *rvs167*, *spa2*, *skt5*, *myo2*, *chs5*, *chs3* and *slt2*) (Table 1; see Text S1). We used the FuncAssociate 2.0 web application [22], capable of identifying GO attributes enriched in lists of genes, to identify GO attributes that were enriched among these congruent genes (Table 2; see Table S4 for the congruent genes associated with each GO attribute). Remarkably, the GO attributes that were given the higher scores were all related to cell wall biogenesis and organization. In fact, the 12 congruent genes were all involved in cell wall biogenesis and organization, suggesting that XopE2 affects these processes in yeast. Interestingly, 8 out of the 12 congruent genes were also involved in endoplasmic reticulum (ER)-nucleus signaling pathway (Table 2).

**Table 1:** Genes identified as congruent to XopE2.

	*slt2*	*chs5*	*smi1*	*swi4*	*cla4*	*swf1*	*rad27*	*nbp2*	SL Overlap^a^	Total SL^b^	p-value	Score^c^
*gas1*	+	+	+	+			+	+	6	16	8.8E-12	11.1
*bni1*	+	+	+	+	+			+	6	18	2.0E-11	10.7
*smi1*	+	+		+	+	+		+	6	22	2.1E-11	10.7
*bem2*	+	+	+	+	+				5	14	1.2E-09	8.9
*bck1*	+	+	+		+	+			5	14	1.2E-09	8.9
*rvs167*	+	+	+	+	+				5	18	5.0E-09	8.3
*spa2*	+		+	+	+				4	7	8.4E-09	8.1
*skt5*	+		+	+	+				4	8	1.7E-08	7.8
*myo2*	+	+	+		+				4	8	1.7E-08	7.8
*chs5*	+		+	+	+				4	10	2.5E-08	7.6
*chs3*	+		+	+	+				4	9	3.0E-08	7.5
*slt2*		+	+	+	+				4	13	8.6E-08	7.1

A plus sign marks an SL interaction between a congruent gene and a deletion strain hypersensitive to XopE2.

a SL Overlap - the number of SL interactions with hypersensitive deletion strains.

b Total SL - the number of SL interactions with the deletion strains in the array.

c Score - Congruence score; the negative logarithm (base 10) of the p-value.

**Table 2:** GO attributes enriched among the genes congruent to XopE2.

Rank	N^a^	X^b^	LOD^c^	p-Value	GO Attribute
1	12	113	2.571	7.3E-15	cellular cell wall organization
2	12	113	2.571	7.3E-15	external encapsulating structure organization
3	12	113	2.571	7.3E-15	cell wall organization
4	12	124	2.523	2.4E-14	cellular cell wall organization or biogenesis
5	12	124	2.523	2.4E-14	cell wall organization or biogenesis
6	11	106	2.086	6.3E-13	site of polarized growth
7	3	8	2.032	1.7E-05	cell wall chitin metabolic process
8	7	29	1.984	2.0E-10	incipient cellular bud site
9	9	56	1.952	7.3E-12	mating projection tip
10	9	59	1.924	1.2E-11	cell projection part
11	3	10	1.897	3.6E-05	cell wall polysaccharide metabolic process
12	8	50	1.844	2.1E-10	ER-nucleus signaling pathway
13	3	11	1.842	4.9E-05	aminoglycan metabolic process
14	3	11	1.842	4.9E-05	chitin metabolic process
15	3	13	1.750	8.5E-05	cell wall macromolecule metabolic process
16	5	31	1.642	1.3E-06	cellular bud tip
17	7	65	1.559	8.1E-08	cellular bud neck
18	9	128	1.530	1.6E-08	intracellular signaling pathway
19	9	136	1.500	2.8E-08	signaling pathway
20	9	136	1.500	2.8E-08	signaling
21	12	768	1.452	1.2E-04	cellular component organization
22	7	89	1.403	7.5E-07	sexual reproduction
23	5	55	1.356	2.5E-05	actin filament-based process
24	7	114	1.281	4.1E-06	reproduction

Results obtained from the FuncAssociate 2.0 web application.

a N - the number of congruent genes that have the GO attribute.

b X - total number of interacting genes covered by our array that have the GO attribute.

c LOD - Logarithm (base 10) of the odds ratio.

### XopE2 causes sensitivity to the cell wall stressing agents caffeine and SDS

**Figure 4:**
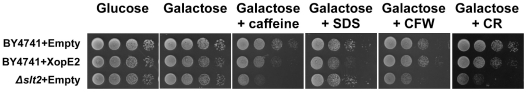
XopE2 causes sensitivity to cell wall stressing agents. The indicated yeast strains containing pGML10, either empty or encoding XopE2, were normalized to OD_600_ = 1.0 and spotted in 10-fold serial dilutions on repressing (2% glucose) and inducing (2% galactose and 1% raffinose) plates with the indicated cell wall stressing agents: caffeine 7 mM; SDS 0.003% (w/v); Congo red (CR) 100 µg ml^−1^; calcofluor white (CFW) 100 µg ml^−1^.

**Figure 5:**
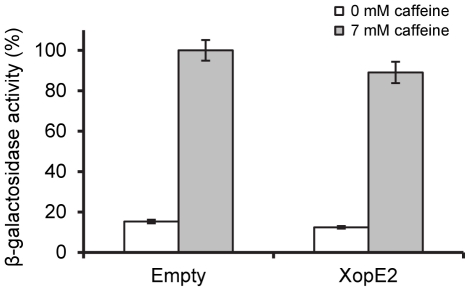
XopE2 does not affect the activation of the CWI pathway. Caffeine-mediated activation of a RLM1-regulated β-galactosidase reporter in yeast expressing XopE2 or an empty expression vector. Activity is reported as percentage of Miller units. 100% activity is set as the activity in yeast treated with 7 mM caffeine in the absence of XopE2. Data represent the mean and standard error (n = 4). The assay was repeated three times with similar results.

The results from the screen prompted us to investigate the effect of XopE2 on the yeast cell wall. We tested the sensitivity of yeast cells expressing XopE2 to a series of cell wall stressing agents, including caffeine, sodium dodecyl sulphate (SDS), calcofluor white and Congo red [25], [26], [27], [28]. As shown in Figure 4, yeast cells expressing XopE2 were sensitive to two cell wall stressing agents, caffeine and SDS, suggesting that XopE2 affected the yeast cell wall. We noted earlier that the *Shigella* T3E OspF affected the yeast cell wall by inhibiting the CWI pathway. Therefore, we were intrigued to test whether XopE2 also affected the activation of this pathway. To this end, we monitored the activity of a *lacZ* reporter driven by a CWI pathway responsive element in response to caffeine, which was previously shown to activate the CWI pathway [29]. As shown in Figure 5, the caffeine-dependent activation of the reporter was not significantly affected by XopE2, suggesting that XopE2 does not directly target the CWI pathway. Hence, it is possible that XopE2 targets a different process related to cell wall biogenesis and organization.

### XopE2 affects the ER stress response

**Figure 6:**
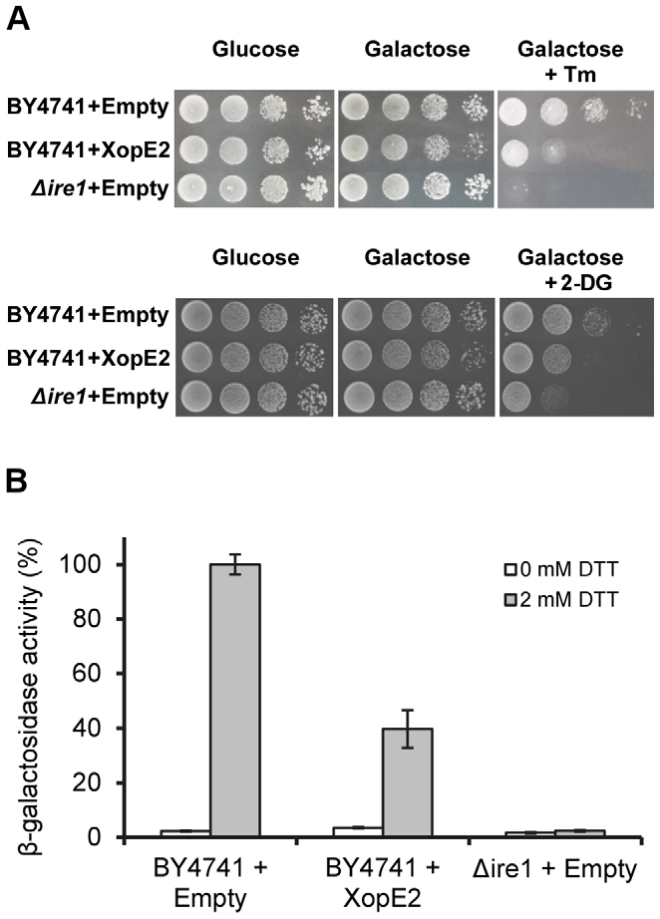
XopE2 affects the ER stress response. **A.** The indicated strains were grown overnight in repressing medium (2% glucose). Cells were then washed, normalized to OD_600_ = 1.0, and spotted in 10-fold serial dilutions on repressing (2% glucose) and inducing (2% galactose and 1% raffinose) plates with 0.165 µg ml^−1^ tunicamycin (Tm; upper panels) or 70 µM 2-deoxy-D-glucose (2-DG; lower panels). **B.** DTT-mediated activation of a UPRE-regulated β-galactosidase reporter. XopE2 was expressed in wild-type cells that were treated or not with 2 mM DTT. The response of the *Δire1* strain was used as a control. Activity is reported as percentage of Miller units. 100% activity is set as the activity in yeast treated with DTT in the absence of XopE2. Data represent the mean and standard error (n = 4). The assays were repeated three times with similar results.

The results of our screen indicated that 8 out of the 12 genes that were identified as congruent to XopE2 were involved in ER-nucleus signaling pathway. This GO attribute was defined by the GO consortium as: “Any series of molecular signals that conveys information from the ER to the nucleus, usually resulting in a change in transcriptional regulation” [30]. The most studied ER-nucleus signaling pathway is the unfolded protein response (UPR), an adaptive stress response that is activated upon sensing an overload of unfolded proteins in the ER [31], [32], [33]. Interestingly, uncompensated ER stress and mutations in the UPR activator Ire1 were shown to cause alteration in cell wall structure, indicating that the UPR is required for cell wall organization and biogenesis [34], [35]. To determine whether XopE2 affects the ER stress response, we tested the sensitivity of yeast cells expressing XopE2 to tunicamycin, a specific inhibitor of N-linked glycosylation in the ER. We found that cells expressing XopE2 were highly sensitive to tunicamycin (Fig. 6A, upper panels). Furthermore, cells expressing XopE2 were also sensitive to 2-deoxy-D-glucose (Fig. 6A, lower panels), an inhibitor of D-mannose incorporation into the dolicholpyrophosphate-bound core oligosaccharide, which causes undergylcosylation of nascent polypeptide chains in the ER [36]. We next tested the ability of yeast cells expressing XopE2 to activate the UPR in response to ER stress. We monitored the expression of a *lacZ* reporter driven by a UPR responsive element in response to the reducing agent dithiothreitol (DTT), which is known to induce ER stress [37]. Remarkably, expression of XopE2 attenuated the activation of the *lacZ* reporter in response to DTT to 40% compared with the activation seen in yeast containing an empty expression vector (Fig. 6B). Altogether, these results suggest that XopE2 affects the ER stress response, linking XopE2 to cell wall organization and biogenesis. To the best of our knowledge, this is the first report of a bacterial effector affecting the ER stress response. The exact mechanism by which XopE2 is operating remains to be elucidated.

## Discussion

In this work, we describe a simple strategy that employs an array of yeast deletion strains to identify cellular processes targeted by virulence proteins. Our strategy is based on the observation that maximal coverage of the yeast SL interaction network does not require the entire collection of null deletion strains. The major advantage of this strategy is that it uses a single 96-well plate instead of over fifty 96-well plates that are used when the entire yeast deletion strain collection is screened. As a proof of concept, we showed that the array of the deletion strains was sufficient to accurately predict a previously identified cellular process targeted by the *Shigella* T3E OspF [12]. Next, we employed the array of the deletion strains to investigate the *Xanthomonas campestris* pv. *vesicatoria* T3E XopE2 for which no cellular target was described. We found that XopE2 was congruent to genes that were all involved in cell wall biogenesis and organization, implying that XopE2 affected these processes. Indeed, we showed that XopE2 caused sensitivity to the cell wall stressing agents caffeine and SDS. Subsequently, we found that XopE2 affected the ER stress response, which is tightly linked to cell wall organization and biogenesis [34], [35]. Thus, we demonstrated the applicability of our approach for studying the functions and targets of bacterial T3Es.

Our approach has several advantages over screening the yeast null deletion strain collection. First, it is simple, convenient and economical, requiring less than 10 days to complete a full screen with relatively few plates. Second, working with a single 96-well plate simplifies the analysis of the results and allows for more repetitions to be made. Importantly, in contrast to previous approaches, our approach does not necessitate the use of a robot, lowering the initial investment required for performing the screen and making it accessible to any laboratory studying virulence proteins that function inside the host cell. Nevertheless, using robotic plating, it is possible to simultaneously screen a large repertoire of effectors, an intriguing possibility in light of the growing number of bacterial proteins identified as effectors.

It should be noted that our approach is suitable for studying bacterial T3Es that target conserved eukaryotic processes found in yeast. It is not expected to yield significant results for T3Es that affect specific processes that cannot be found in yeast.

The hypersensitive deletion strains identified in the screen can be used in additional ways. First, the hypersensitive deletion strains can be used to screen for genes, which upon over-expression, suppress the growth inhibition phenotype caused by the T3Es. Finding such suppressors can assist in identifying the cellular processes that are targeted by the T3Es. Second, the hypersensitive deletion strains can be used to classify T3Es of various pathogens into functional groups, laying the foundation for future study of “functional effector families”.

Several factors affected our selection of the expression vector. Our system employs the GAL1/10 promoter, a strong promoter whose activity is regulated by the carbon source in the medium. An important feature of the GAL1/10 promoter is that it does not require the use of modified yeast strains, which simplified the construction of the array. The use of an inducible expression vector enabled us to perform the transformation step under conditions in which the expression of the bacterial effector is repressed, grow the transformed cells to saturation and only then spot them on inducing and repressing plates. In this way, we eliminated the effect of variations in transformation efficiency between deletion strains. Another important factor that influenced our selection of the expression vector was the number of copies of the effector gene in the cell. It was previously suggested that high-level expression of the bacterial effector (when using a 2 micron vector) might result in non-specific activity of the effector [18]. Our system uses a centromere-containing vector to obtain low-level expression of the bacterial effector and thus to increase the specificity of the assay. The expression vector that we use also contains a single myc tag, which allows to monitor the expression of the effector in the cell. The tag is fused to the C-terminal tail of the effector and owing to its short size it is not likely to affect the expression or the function of the effector.

Our approach requires the transformation of the array of deletion strains with the vector encoding the bacterial effector. One way to avoid this step is to transform a single yeast strain with the vector encoding the bacterial effector, and by mating and meiosis to transfer the vector to the deletion strains. However, this approach, known as the synthetic genetic array (SGA) methodology [38], [39], is much slower, requiring at least two weeks, not including the time required for the transformation of the starting strain [40], [41]. Nevertheless, the SGA methodology should be considered when a large number of bacterial T3Es are screened simultaneously, ideally with the aid of a robot. Another mating-based approach, which is expected to be much faster than the SGA methodology, is called selective ploidy ablation (SPA) [42]. This approach employs a universal plasmid donor strain that contains conditional centromeres on every chromosome. The plasmid-bearing donor strain is mated to a recipient, followed by removal of all donor-strain chromosomes, producing a haploid strain containing the transferred plasmid. One limitation of the SPA approach is that chromosomes destabilization requires growth on galactose, which induces the expression of the bacterial effector in our system.

Finally, although we concentrated our work on bacterial T3Es, our approach can be easily employed to study other types of virulence proteins that function inside host cells, such as bacterial type IV and type VI secreted effectors, fungal effectors and viral proteins. In conclusion, the approach presented in this work provides an excellent platform for studying the functions and cellular targets of bacterial effectors and other virulence proteins.

## Materials and Methods

### Synthetic lethal interaction and phenotypic data

SL interactions and phenotypic data were extracted from the *Saccharomyces* Genome Database (http://www.yeastgenome.org, downloaded on 19 April 2011). It was assumed that all SL interactions were symmetric. The database contains 10,438 unique SL interactions between 2,795 genes (435 genes interact only with genes marked in the *Saccharomyces* Genome Database as inviable and were not taken into account).

### Media and bacterial and yeast strains

Bacteria used in this study are *E. coli* DH12S. Bacteria were grown in Luria-Bertani broth supplemented with 100 µg/ml ampicillin at 37°C [43]. Yeast strains used in this study are BY4741 (*MATa his3Δ1 leu2Δ0 met15Δ0 ura3Δ0*) and the BY-series deletion strains [44] of the genes listed in Table S1. Yeast were grown at 30°C in YPD medium (1% yeast extract, 2% peptone, 2% glucose) or in selective synthetic complete media lacking uracil and/or leucine to maintain plasmids, and supplemented with 2% glucose or 2% galactose and 1% raffinose as carbon sources [45].

### 96-well microtiter plate yeast transformation protocol

Glycerol stocks of the yeast haploid deletion strains were plated onto YPD agar and incubated at 30°C for 2 days. Single colonies were picked into a round-bottom 96-well microtiter plate containing 150 µl of YPD in each well. The microtiter plate was incubated overnight at 30°C. Next, the microtiter plate was centrifuged for 5 minutes at 700 *g* and the supernatant was removed by a single shake of the plate into a large sink. Cells were resuspended with 100 µl/well DDW using an 8-channel multi-pipettor, and 25 µl from each well were transferred to a new round-bottom 96-well microtiter plate containing 75 µl/well DDW. The microtiter plate was centrifuged for 5 minutes at 700 *g* and the supernatant was removed. Cells were resuspended in 50 µl of a freshly prepared transformation mix (0.3 M LiAc pH∼7.5, 1 mg/ml boiled single strand Salmon sperm DNA, 4 ng/µl plasmid DNA) [46]. After resuspension, 100 µl of 50% (w/v) PEG 3350 were added to each well with truncated tips and mixed with the transformation mix. The microtiter plate was then placed in a plastic bag and incubated at 42°C for 2 hours with constant shaking. After incubation, 10 µl/well DMSO were added and the plate was placed again in a plastic bag and incubated at 42°C for 30 minutes with constant shaking. Following incubation, the plate was centrifuged at 1500 *g* for 10 minutes and the supernatant removed as described above. The plate was washed 3 times by addition of 100 µl/well DDW followed by centrifugation at 700 *g* for 5 minutes and removal of the supernatant to dispose of residual PEG 3350. Finally, cells were resuspended in 10 µl/well DDW, and transformations were spotted onto selective synthetic complete media lacking leucine and supplemented with 2% glucose [45]. Plates were incubated at 30°C for 2–3 days.

### Identification of processes targeted by XopE2 using the array of deletion strains

The yeast strains of the array were transformed with pGML10 vector either empty or encoding a galactose inducible XopE2 [47]. Transformed cells were spotted in quadruplicates on both repressing (2% glucose) and inducing (2% galactose and 1% raffinose) media in Omni trays (Nunc, http://www.nuncbrand.com) and were allowed to grow for 2–3 days. The spots from three biological repetitions were digitally quantified using the Otsu's method [48] (see Fig. S3 for a summary of the quantification procedure). The resulting values were used to determine which deletion strains were hypersensitive to the expression of XopE2 (see Figs. S4 and S5 for examples of the data analysis). The hypersensitive deletion strains were analyzed to identify congruent genes, using yeast SL interactions data extracted from the *Saccharomyces* Genome Database. The list of congruent genes was used to identify potential cellular targets of XopE2, using the FuncAssociate 2.0 web application (http://llama.mshri.on.ca/funcassociate/) [22]. Similar analysis was performed for the *Shigella* T3E OspF. The various procedures are described in Text S1.

### Spotting assays

BY4741 yeast strains, either wild-type or from the deletion strain collection, were transformed with pGML10, either empty or encoding XopE2. Cells were grown overnight in repressing medium (2% glucose), washed and normalized to OD_600_ = 1.0, and 10-fold or 5-fold serial dilutions, as indicated, were spotted onto repressing and inducing (2% galactose and 1% raffinose) media with or without the indicated stressing agents. Pictures were taken after 2–3 days of growth at 30°C.

### *LacZ* reporter activation assays

To determine activation of the CWI pathway, yeast containing the RLM1-regulated *lacZ* reporter [12], [29] and the pGML10 vector either empty or encoding XopE2 [47] were grown overnight in selective media containing glucose (2%). Cultures were washed, diluted and grown to OD_600_ = 0.5–0.8 in selective media containing galactose (2%) and raffinose (1%). Cultures were then supplemented with 7 mM caffeine or the equivalent volume of water, and incubated at 30°C for 4 hours. After incubation, cells were collected and subjected to a β-galactosidase activity assay.

To determine activation of the UPR, yeast containing a UPRE-regulated *lacZ* reporter [49] and the pGML10 vector either empty or encoding XopE2 [47] were grown overnight in selective media containing glucose (2%). Cultures were washed, diluted and grown to OD_600_ = 0.5–0.8 in selective media containing galactose (2%) and raffinose (1%). Cultures were then supplemented with 2 mM DTT or the equivalent volume of water, and incubated at 30°C for 4 hours. After incubation, cells were collected and subjected to a β-galactosidase activity assay. Quantitative assays for β-galactosidase activity were performed as described [50], [51].

## Supporting Information

Text S1 **Supporting methods.** The supporting methods include additional information on the construction of the 96-well plate, the screen for deletion strains hypersensitive to bacterial type III effectors, analysis of the results from the screens, identification of congruent genes, and identification of possible cellular targets using FuncAssociate 2.0.
(PDF)

Figure S1 **Location of the various strains in the 96-well plate.** The *Δsec22* strain (marked with an asterisk) was removed from the analysis due to poor growth in several repetitions.
(TIF)

Figure S2 **Validation of the hypersensitivity of the deletion strains to XopE2.** The indicated yeast strains containing pGML10, either empty or encoding XopE2, were normalized to OD_600_ = 1.0 and spotted in 5-fold serial dilutions on repressing (2% glucose) and inducing (2% galactose and 1% raffinose) plates.
(TIF)

Figure S3 **Quantification of the results from the screen.** **A,** The plates are scanned and the images are edited to remove margins, scratches and small stains. **B,** The images are partitioned into a 16×24 grid of squares, each containing a single spot. **C,** The images are converted to binary images by computing the global image threshold (Otsu's method). **D,** The white pixels in each square are counted and are saved for further analysis.
(TIF)

Figure S4 **Calculation of the sensitivity of** ***Δswf1*** **to XopE2.** **A,** Inducing/repressing ratio is calculated by dividing the average number of white pixels of the quadruplicates on the inducing plate by the average number of white pixels of the quadruplicates on the repressing plate. The inducing/repressing ratio of the wild-type strain is the average of all the transformations of the wild-type strain. **B,** Growth ratio is calculated by dividing the inducing/repressing ratio of each strain by the inducing/repressing ratio of the wild-type strain. **C,** Relative growth ratio is calculated by dividing the growth ratios of each deletion strain containing XopE2 by the average of the growth ratio of the deletion strain containing an empty vector.
(TIF)

Figure S5 ***Δbim1*** **is not hypersensitive to XopE2.** See Figure S4 for description of the calculation steps.
(TIF)

Table S1 **The list of deletion strains used to construct the array.**
(PDF)

Table S2 **Genes identified as congruent to OspF.**
(PDF)

Table S3 **GO attributes enriched among the genes congruent to OspF.**
(PDF)

Table S4 **GO attributes enriched among the genes congruent to XopE2 (including the congruent genes).**
(PDF)
