# Supplementary material for: A Simple Yeast-Based Strategy to Identify Host Cellular Processes Targeted by Bacterial Effector Proteins
Source: PLoS One. 2011 Nov 15;6(11):e27698. doi: 10.1371/journal.pone.0027698 (PMC3216995; doi:10.1371/journal.pone.0027698)
Supplement: Text S1 — Supporting methods. The supporting methods include additional information on the construction of the 96-well plate, the screen for deletion strains hypersensitive to bacterial type III effectors, analysis of the results from the screens, identification of congruent genes, and identification of possible cellular targets using FuncAssociate 2.0. (PDF) [file pone.0027698.s001.pdf]

Text S1. Supporting methods

Construction of the 96-well plate

We corroborated the results of the selection algorithm described in the main text using the Genetic Algorithm, an evolution-inspired optimization technique, which we employed in the past to determine the rate constants of chemical-kinetic models [1,2,3]. The adjustable parameters in this case were the 90 deletion strains selected randomly out of the list of viable deletion strains. We discarded the deletion strains with no SL interactions, to increase the efficiency of the algorithm. The fitness function, which was used in the optimization process, was defined as the sum of the number of genes covered by the selected deletion strains plus the number of genes covered by two or more of the selected deletion strains.

Due to poor growth, several deletion strains (*Δcdc40, Δcog1, Δget1, Δget2, Δhpr1, Δknh1, Δpho85, Δrad6, Δrvs167, Δtho2* and *Δyng2*) had to be substituted by other deletion strains that covered similar SL interactions or by copies of the wild-type strain. The final 96-well plate contained 92 deletion strains and 4 copies of the wild-type strain (Fig. S1 and Table S1). The *Δsec22* strain was later removed from the analysis due to poor growth in several repetitions.

A screen for deletion strains hypersensitive to bacterial type III effectors

The deletion strains (Euroscarf, http://web.uni-frankfurt.de/fb15/mikro/euroscarf/index.html) listed in Table S1 were transformed with pGML10 either empty or encoding XopE2 using the 96-well microtiter plate transformation protocol described in the main text. Transformants were grown on selective synthetic complete solid media lacking leucine (hereafter called selective media) supplemented with 2% glucose. Transformed colonies were picked into a round-bottom microtiter plate containing 200 µl of selective media supplemented with 2% glucose in each well, and grown at 30°C overnight. In the next morning, the microtiter plate was centrifuged and supernatant was removed. Cells were resuspended in 100 µl/well of

DDW and diluted 10-fold into a new round-bottom microtiter plate containing 90 µl/well of DDW. Using a 1.58 mm 96-floating pin replicator (VP 408FP6, V&P Scientific, http://www.vp-scientific.com/index.html) and a Colony copier (VP 380, V&P Scientific), transformations were plated in quadruplicates to create a 384-spots array on inducing (2% galactose and 1% raffinose) and repressing (2% glucose) selective media in Omni trays (Nunc, http://www.nuncbrand.com). The repressing and inducing plates were incubated at 30°C for 2 and 3 days, respectively. Each screen was repeated three times.

Analysis of the results from the screens

The quantification of the spots was carried out according to the procedure summarized in Figure S3. First, the plates were scanned and the resulting images were edited to remove margins, scratches and small stains. This step was performed manually using an editing program. Then, the images were partitioned into a 16x24 grid of squares, each containing a single spot. The horizontal and vertical lines were positioned so clashes between the lines and the spots would be minimized. Afterward, the images were converted to binary images by computing the global image threshold according to Otsu's method implemented in Matlab [4]. The output binary images had values of 0 (black) for all pixels in the input image with luminance less than the threshold level and 1 (white) for all other pixels. Finally, the white pixels in each square were counted and saved for further analysis. The partitioning of the images, the conversion to binary images and the counting of white pixels were performed automatically using code written in Matlab.

The following calculations were performed in order to evaluate the sensitivity of the deletion strains to the effector XopE2. The inducing/repressing ratio of each transformation was calculated by dividing the average number of white pixels of the quadruplicates on the inducing plate by the average number of white pixels of the quadruplicates on the repressing plate. The inducing/repressing ratio of the wild-type strain was the average of all the transformations of the wild-type strain. To take into consideration the effect of the inducing

conditions, the inducing/repressing ratio of each strain was divided by the inducing/repressing ratio of the wild-type strain, yielding the growth ratio. All of these calculations were performed for transformations containing an empty vector and transformations containing the vector encoding XopE2. The growth ratios of each deletion strain containing XopE2 were divided by the average of the growth ratio of the deletion strain containing an empty vector, yielding the relative growth ratio. The relative growth ratio served as an indicator of the sensitivity of a deletion strain to the bacterial effector. A deletion strain was defined as hypersensitive to XopE2 if the relative growth ratio of the strain was lower than 50% in at least two of the three biological repetitions.

The calculation of the sensitivity of *Δswf1* to XopE2 is given as an example for the data analyses (Fig. S4). First, the inducing/repressing ratios of the transformations were calculated from the quantification of the spots (Fig. S4A). The wild-type transformations exhibited inducing/repressing ratios between 84.7% and 98.1% when they contained the empty vector, and growth ratios between 58.4% and 87.7% when they contained the vector encoding XopE2. The *Δswf1* transformations showed growth ratios between 50.3% and 86.8% when they contained an empty vector and growth ratios between 0.6% and 10.2% when they contained the vector encoding XopE2. The inducing/repressing ratios indicated that *Δswf1* transformations were more sensitive to the inducing conditions compared to the wild-type transformations. It also indicated that XopE2 had a mild effect on the growth of the wild-type transformations. To consider the effect of the inducing conditions, the inducing/repressing ratios were divided by the values of the corresponding wild-types of each biological repetition. The growth ratios of the *Δswf1* transformations were between 59.4% and 88.5% when they contained an empty vector and between 1.0% and 11.7% when they contained the vector encoding XopE2 (Fig. S4B). The average growth ratio of the *Δswf1* transformations containing an empty vector was 73.6%. The relative growth ratios of *Δswf1* in each biological repetition, indicating on the sensitivity of *Δswf1* to XopE2, were calculated by dividing the growth ratios of the *Δswf1* transformations containing the vector encoding XopE2 by the

average growth ratio of the *Δswf1* transformations containing an empty vector (Fig. S4C). In each of the three biological repetitions the relative growth ratio of the *Δswf1* strain was lower than 50%. Thus, according to the criteria presented earlier the *Δswf1* strain was hypersensitive to the effector XopE2. The calculation of *Δbim1* is given as an example for a deletion strain which is not hypersensitive to XopE2 (Fig. S5).

Identification of congruent genes

We now discuss the identification of yeast genes congruent to the effector XopE2. As discussed in the main text, genes were defined as congruent to a bacterial effector, if their sets of SL interactions overlapped with the deletion strains found to be hypersensitive to the effector. To identify genes congruent to XopE2, we first compiled a list of the genes that were synthetic lethal with any of the eight deletion strains hypersensitive to XopE2 (*Δslt2*, *Δchs5*, *Δsmi1*, *Δswi4*, *Δcla4*, *Δswf1*, *Δrad27* and *Δnbp2*). For each of the genes, we counted the number of interactions with the eight hypersensitive genes (SL overlap) and the number of possible interactions with our array of deletion strains (Total SL). We used these numbers to calculate the probability of each gene sharing SL interactions with XopE2 (thoroughly discussed in [5,6]). The p-value, which was derived from the hypergeometric distribution, was converted to congruence score, defined as the negative logarithm (base 10) of the p-value [5]. The Bonferroni correction defined an alpha value of 0.05/1,624 ≈ 3.08 e-5 which was equivalent to a congruence score of ~4.51. To increase the significance of the GO attributes identified based on the congruent genes we selected a more stringent threshold of 7.0 [5]. The entire process was performed automatically using queries and modules created in Microsoft Access.

Identification of possible cellular targets using FuncAssociate 2.0

The FuncAssociate 2.0 web application (http://llama.mshri.on.ca/funcassociate/) [7], capable of identifying GO attributes enriched in lists of genes or proteins, was used to identify possible cellular processes targeted by bacterial T3Es. The yeast genes identified as congruent

to XopE2, representing possible cellular processes targeted by the bacterial effector, were given as an input for the enrichment analysis performed by the FuncAssociate application. In addition, a user-specified genespace, which included the 1,624 genes covered by our array of deletion strains, was provided to increase the accuracy of the enrichment analysis. The results produced by the FuncAssociate application were ordered by the logarithm (base 10) of the odds ratio (LOD) values.

References

1. Bosis E, Nachliel E, Cohen T, Takeda Y, Ito Y, et al. (2008) Endoplasmic reticulum glucosidase II is inhibited by its end products. Biochemistry 47: 10970-10980.

2. Moscovitch D, Noivirt O, Mezer A, Nachliel E, Mark T, et al. (2004) Determination of a unique solution to parallel proton transfer reactions using the genetic algorithm. Biophys J 87: 47-57.

3. Sengupta P, Bosis E, Nachliel E, Gutman M, Smith SO, et al. (2009) EGFR juxtamembrane domain, membranes, and calmodulin: kinetics of their interaction. Biophys J 96: 4887-4895.

4. Otsu N (1979) Threshold Selection Method from Gray-Level Histograms. Ieee Transactions on Systems Man and Cybernetics 9: 62-66.

5. Ye P, Peyser BD, Pan X, Boeke JD, Spencer FA, et al. (2005) Gene function prediction from congruent synthetic lethal interactions in yeast. Mol Syst Biol 1: 2005 0026.

6. Kramer RW, Slagowski NL, Eze NA, Giddings KS, Morrison MF, et al. (2007) Yeast functional genomic screens lead to identification of a role for a bacterial effector in innate immunity regulation. PLoS Pathog 3: e21.

7. Berriz GF, Beaver JE, Cenik C, Tasan M, Roth FP (2009) Next generation software for functional trend analysis. Bioinformatics 25: 3043-3044.
