# Supplementary figures and images for: A Simple Yeast-Based Strategy to Identify Host Cellular Processes Targeted by Bacterial Effector Proteins
Source: PLoS One. 2011 Nov 15;6(11):e27698. doi: 10.1371/journal.pone.0027698 (PMC3216995; doi:10.1371/journal.pone.0027698)

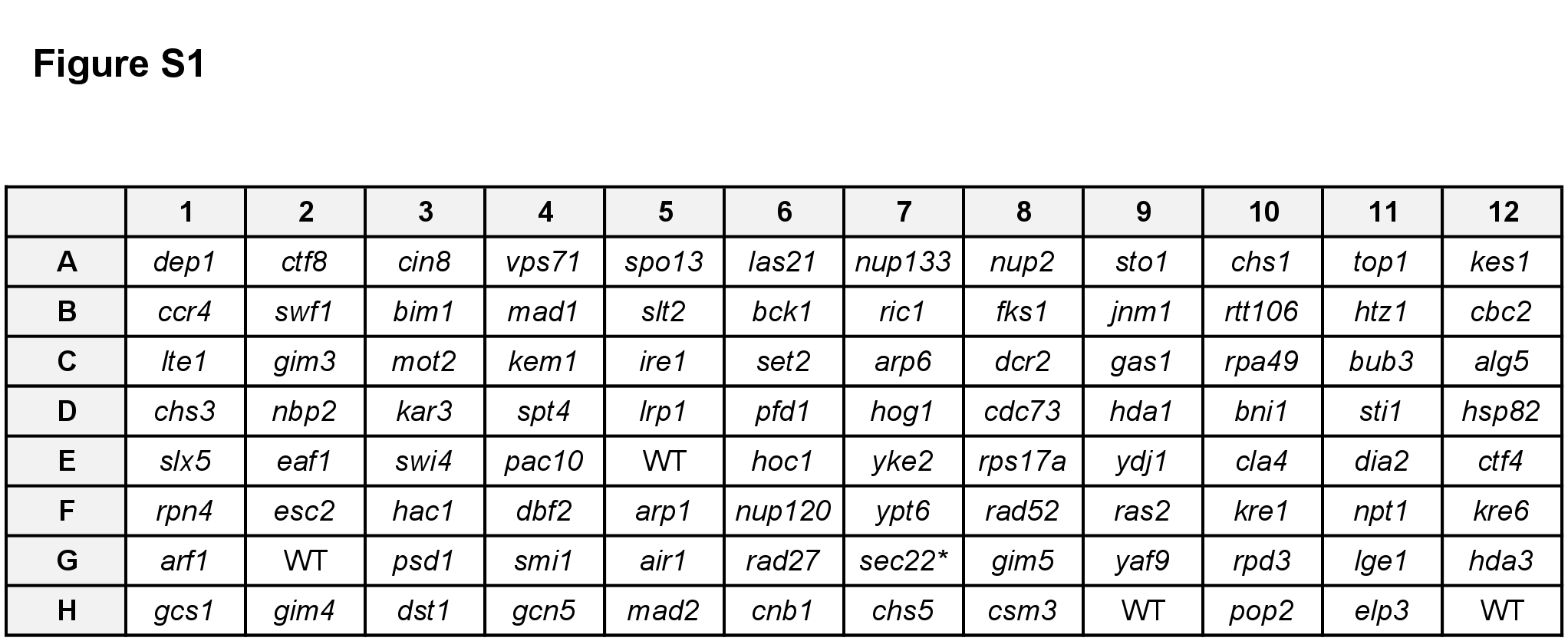

Supplement: Figure S1 — Location of the various strains in the 96-well plate. The Δsec22 strain (marked with an asterisk) was removed from the analysis due to poor growth in several repetitions. (TIF) [file pone.0027698.s002.tif]

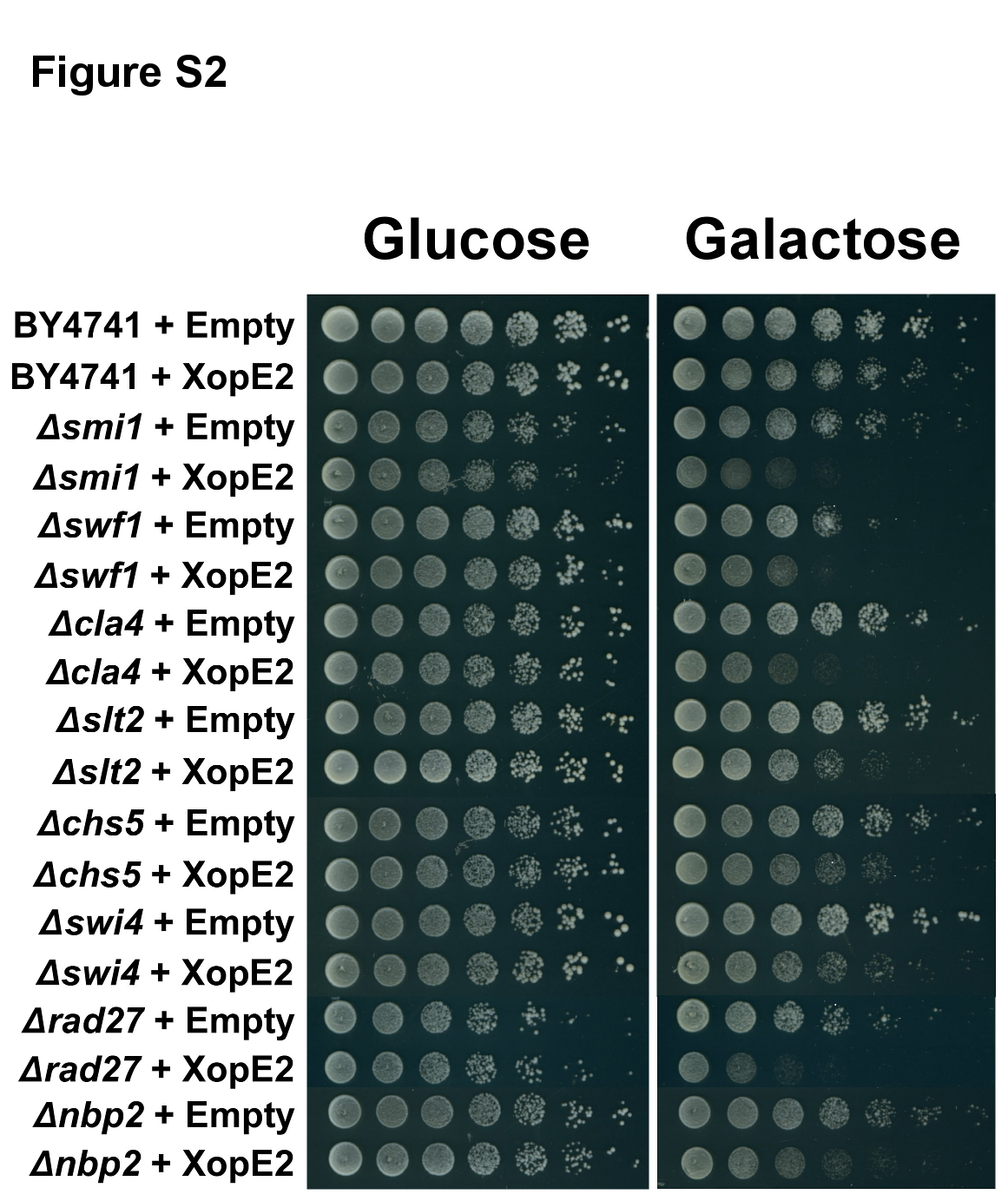

Supplement: Figure S2 — Validation of the hypersensitivity of the deletion strains to XopE2. The indicated yeast strains containing pGML10, either empty or encoding XopE2, were normalized to OD600 = 1.0 and spotted in 5-fold serial dilutions on repressing (2% glucose) and inducing (2% galactose and 1% raffinose) plates. (TIF) [file pone.0027698.s003.tif]

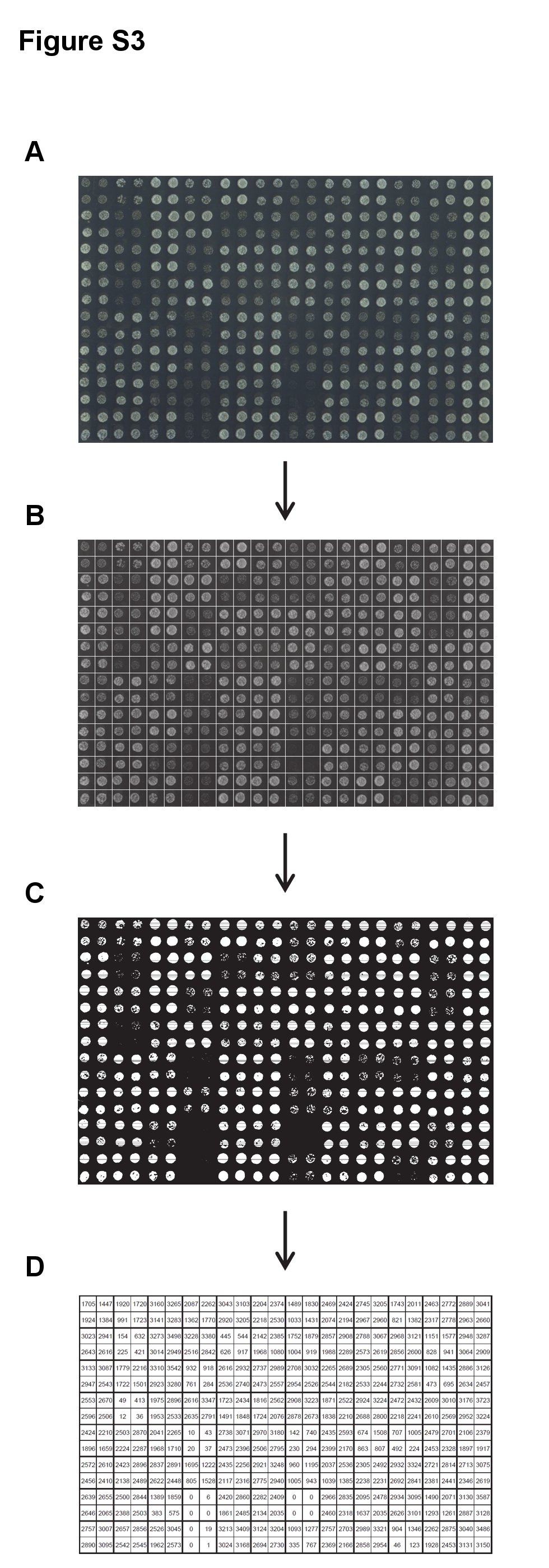

Supplement: Figure S3 — Quantification of the results from the screen. A, The plates are scanned and the images are edited to remove margins, scratches and small stains. B, The images are partitioned into a 16×24 grid of squares, each containing a single spot. C, The images are converted to binary images by computing the global image threshold (Otsu's method). D, The white pixels in each square are counted and are saved for further analysis. (TIF) [file pone.0027698.s004.tif]

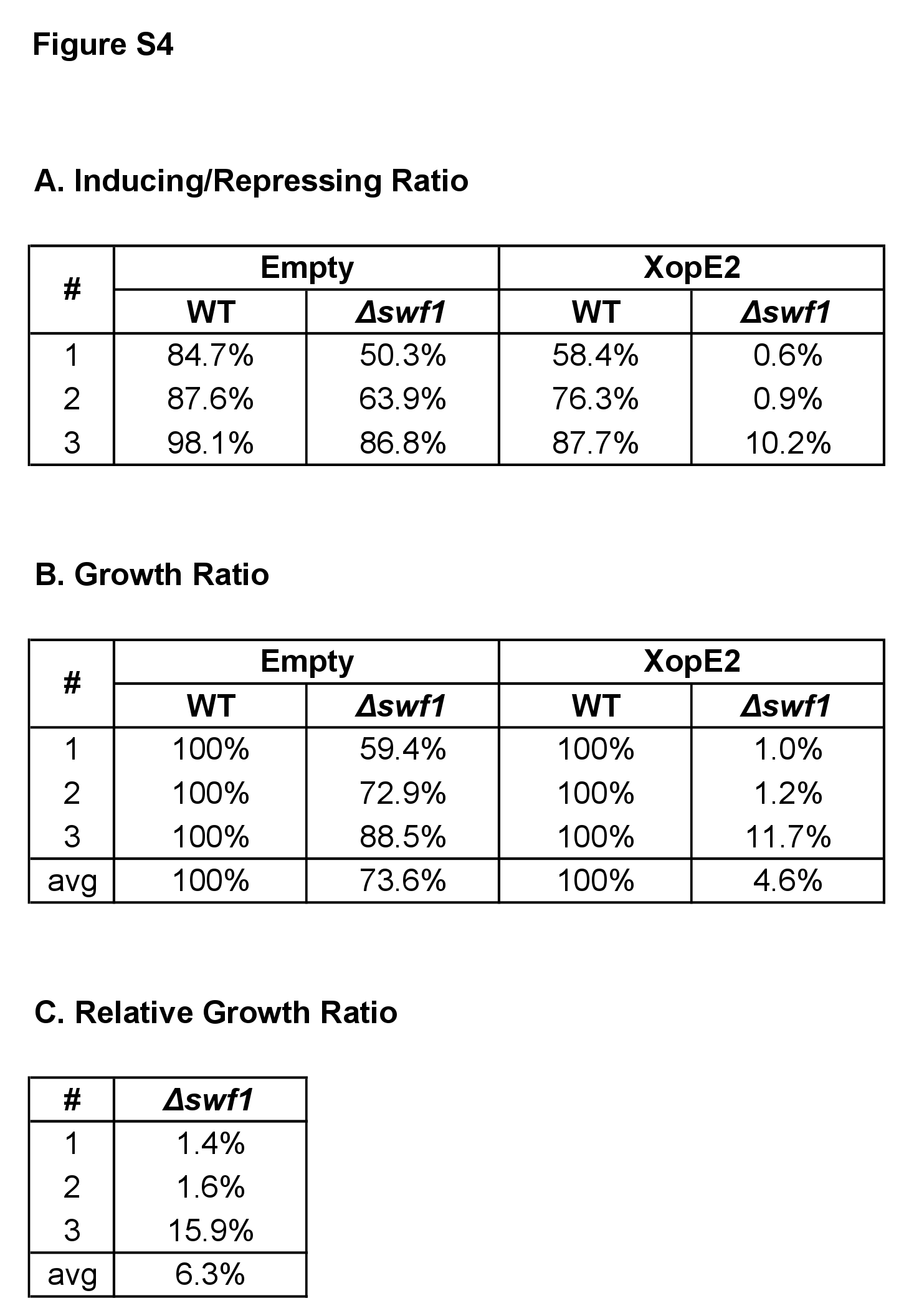

Supplement: Figure S4 — Calculation of the sensitivity of Δswf1 to XopE2. A, Inducing/repressing ratio is calculated by dividing the average number of white pixels of the quadruplicates on the inducing plate by the average number of white pixels of the quadruplicates on the repressing plate. The inducing/repressing ratio of the wild-type strain is the average of all the transformations of the wild-type strain. B, Growth ratio is calculated by dividing the inducing/repressing ratio of each strain by the inducing/repressing ratio of the wild-type strain. C, Relative growth ratio is calculated by dividing the growth ratios of each deletion strain containing XopE2 by the average of the growth ratio of the deletion strain containing an empty vector. (TIF) [file pone.0027698.s005.tif]

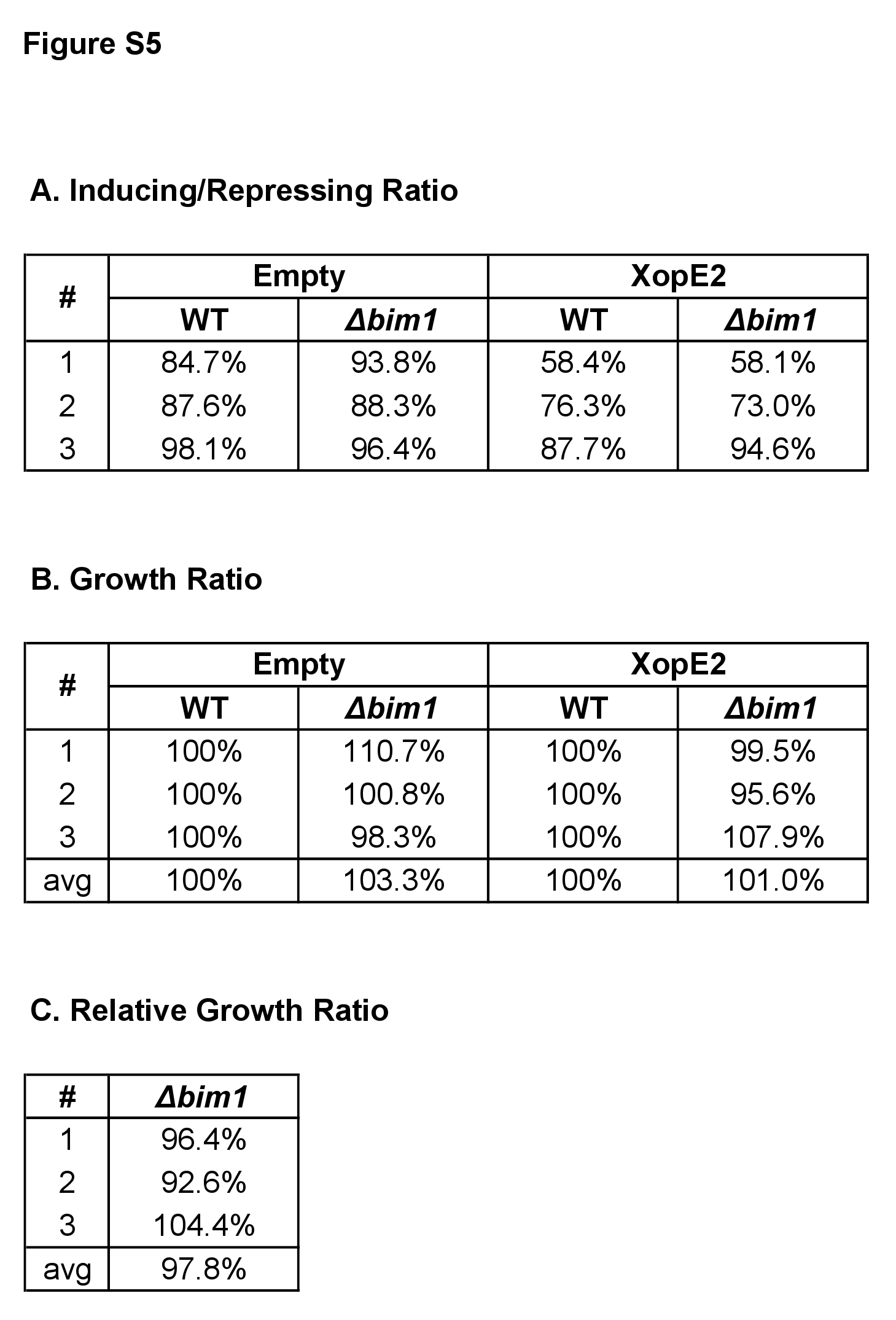

Supplement: Figure S5 — Δbim1 is not hypersensitive to XopE2. See Figure S4 for description of the calculation steps. (TIF) [file pone.0027698.s006.tif]
