# Supplementary material for: A Simple Yeast-Based Strategy to Identify Host Cellular Processes Targeted by Bacterial Effector Proteins
Source: PLoS One. 2011 Nov 15;6(11):e27698. doi: 10.1371/journal.pone.0027698 (PMC3216995; doi:10.1371/journal.pone.0027698)
Supplement: Table S1 — The list of deletion strains used to construct the array. (PDF) [file pone.0027698.s007.pdf]

Table S1. The list of deletion strains used to construct the array

#	Systematic Name	Standard Name	#	Systematic Name	Standard Name
1	*YAL013W*	*DEP1*	47	*YLR200W*	*YKE2*
2	*YAL021C*	*CCR4*	48	*YLR262C*	*YPT6*
3	*YAL024C*	*LTE1*	49	*YLR268W*	*SEC22*
4	*YBR023C*	*CHS3*	50	*YLR330W*	*CHS5*
5	*YDL013W*	*SLX5*	51	*YLR335W*	*NUP2*
6	*YDL020C*	*RPN4*	52	*YLR342W*	*FKS1*
7	*YDL192W*	*ARF1*	53	*YLR361C*	*DCR2*
8	*YDL226C*	*GCS1*	54	*YLR418C*	*CDC73*
9	*YDR126W*	*SWF1*	55	*YML024W*	*RPS17A*
10	*YDR162C*	*NBP2*	56	*YML032C*	*RAD52*
11	*YDR359C*	*EAF1*	57	*YML041C*	*VPS71*
12	*YDR363W*	*ESC2*	58	*YML094W*	*GIM5*
13	*YEL003W*	*GIM4*	59	*YMR048W*	*CSM3*
14	*YEL061C*	*CIN8*	60	*YMR125W*	*STO1*
15	*YER016W*	*BIM1*	61	*YMR294W*	*JNM1*
16	*YER068W*	*MOT2*	62	*YMR307W*	*GAS1*
17	*YER111C*	*SWI4*	63	*YNL021W*	*HDA1*
18	*YFL031W*	*HAC1*	64	*YNL064C*	*YDJ1*
19	*YGL043W*	*DST1*	65	*YNL098C*	*RAS2*
20	*YGL086W*	*MAD1*	66	*YNL107W*	*YAF9*
21	*YGL173C*	*KEM1*	67	*YNL153C*	*GIM3*
22	*YGR063C*	*SPT4*	68	*YNL169C*	*PSD1*
23	*YGR078C*	*PAC10*	69	*YNL192W*	*CHS1*
24	*YGR092W*	*DBF2*	70	*YNL206C*	*RTT106*
25	*YGR229C*	*SMI1*	71	*YNL248C*	*RPA49*
26	*YGR252W*	*GCN5*	72	*YNL271C*	*BNI1*
27	*YHR014W*	*SPO13*	73	*YNL298W*	*CLA4*
28	*YHR030C*	*SLT2*	74	*YNL322C*	*KRE1*
29	*YHR079C*	*IRE1*	75	*YNL330C*	*RPD3*
30	*YHR081W*	*LRP1*	76	*YNR052C*	*POP2*
31	*YHR129C*	*ARP1*	77	*YOL006C*	*TOP1*
32	*YHR191C*	*CTF8*	78	*YOL012C*	*HTZ1*
33	*YIL079C*	*AIR1*	79	*YOR026W*	*BUB3*
34	*YJL030W*	*MAD2*	80	*YOR027W*	*STI1*
35	*YJL062W*	*LAS21*	81	*YOR080W*	*DIA2*
36	*YJL095W*	*BCK1*	82	*YOR209C*	*NPT1*
37	*YJL168C*	*SET2*	83	*YPL055C*	*LGE1*
38	*YJL179W*	*PFD1*	84	*YPL086C*	*ELP3*
39	*YJR075W*	*HOC1*	85	*YPL145C*	*KES1*
40	*YKL057C*	*NUP120*	86	*YPL178W*	*CBC2*
41	*YKL113C*	*RAD27*	87	*YPL227C*	*ALG5*
42	*YKL190W*	*CNB1*	88	*YPL240C*	*HSP82*
43	*YKR082W*	*NUP133*	89	*YPR135W*	*CTF4*
44	*YLR039C*	*RIC1*	90	*YPR141C*	*KAR3*
45	*YLR085C*	*ARP6*	91	*YPR159W*	*KRE6*
46	*YLR113W*	*HOG1*	92	*YPR179C*	*HDA3*

All strains belong to the BY-series with a KanMX cassette inserted into the specified locus.
