# Supplementary material for: A Simple Yeast-Based Strategy to Identify Host Cellular Processes Targeted by Bacterial Effector Proteins
Source: PLoS One. 2011 Nov 15;6(11):e27698. doi: 10.1371/journal.pone.0027698 (PMC3216995; doi:10.1371/journal.pone.0027698)
Supplement: Table S2 — Genes identified as congruent to OspF. (PDF) [file pone.0027698.s008.pdf]

Table S2. Genes identified as congruent to OspF

	ccr4	***smi1***	***las21***	***fks1***	***gim5***	***gas1***	***bni1***	***kre1***	***pop2***	**SL Overlap**[a]	**Total SL**[b]	**p-value**	**Score**[c]
swi4	+	+		+		+	+		+	6	14	9.9E-12	11.0
rvs167	+	+		+	+			+	+	6	18	6.1E-11	10.2
pkc1		+	+	+		+	+			5	8	7.5E-11	10.1
jnm1		+		+	+	+	+			5	13	1.7E-09	8.8
chs6		+	+	+		+				4	7	1.5E-08	7.8
erg2	+						+	+	+	4	7	1.5E-08	7.8
skt5		+		+		+	+			4	8	3.0E-08	7.5
nbp2		+			+	+	+			4	8	3.0E-08	7.5
mms22	+	+		+					+	4	9	5.4E-08	7.3
chs7		+		+		+	+			4	9	5.4E-08	7.3
chs3		+		+		+	+			4	9	5.4E-08	7.3
cnb1		+		+		+		+		4	9	5.4E-08	7.3
chs5		+		+		+	+			4	10	9.0E-08	7.0

A plus sign marks an SL interaction between a congruent gene and a deletion strain hypersensitive to OspF.

[a]SL Overlap - the number of SL interactions with hypersensitive deletion strains.

[b]Total SL - the number of SL interactions with the deletion strains in the array.

[c]Score - Congruence score; the negative logarithm (base 10) of the p-value.
