# Supplementary material for: A Simple Yeast-Based Strategy to Identify Host Cellular Processes Targeted by Bacterial Effector Proteins
Source: PLoS One. 2011 Nov 15;6(11):e27698. doi: 10.1371/journal.pone.0027698 (PMC3216995; doi:10.1371/journal.pone.0027698)
Supplement: Table S3 — GO attributes enriched among the genes congruent to OspF. (PDF) [file pone.0027698.s009.pdf]

Table S3. GO attributes enriched among the genes congruent to OspF

Rank	N[a]	X[b]	LOD[c]	p-Value	GO Attribute
1	5	8	2.473	7.6E-10	cell wall chitin metabolic process
2	4	7	2.338	8.6E-08	cell wall chitin biosynthetic process
3	4	7	2.338	8.6E-08	fungal-type cell wall chitin biosynthetic process
4	4	7	2.338	8.6E-08	fungal-type cell wall polysaccharide biosynthetic process
5	4	7	2.338	8.6E-08	cell wall polysaccharide biosynthetic process
6	5	10	2.276	3.4E-09	cell wall polysaccharide metabolic process
7	5	11	2.204	6.2E-09	aminoglycan metabolic process
8	5	11	2.204	6.2E-09	chitin metabolic process
9	5	13	2.087	1.7E-08	cell wall macromolecule metabolic process
10	4	10	2.068	5.1E-07	aminoglycan biosynthetic process
11	4	10	2.068	5.1E-07	chitin biosynthetic process
12	4	12	1.951	1.2E-06	cellular cell wall macromolecule metabolic process
13	4	12	1.951	1.2E-06	cell wall macromolecule biosynthetic process
14	4	12	1.951	1.2E-06	cellular component macromolecule biosynthetic process
15	4	14	1.859	2.4E-06	cellular polysaccharide biosynthetic process
16	4	17	1.749	5.6E-06	polysaccharide biosynthetic process
17	5	26	1.680	8.3E-07	polysaccharide metabolic process
18	7	50	1.619	2.5E-08	ER-nucleus signaling pathway
19	4	22	1.611	1.7E-05	cellular polysaccharide metabolic process
20	4	28	1.487	4.6E-05	cellular carbohydrate biosynthetic process
21	9	113	1.484	1.6E-08	cellular cell wall organization
22	9	113	1.484	1.6E-08	external encapsulating structure organization
23	9	113	1.484	1.6E-08	cell wall organization
24	9	124	1.437	3.7E-08	cellular cell wall organization or biogenesis
25	9	124	1.437	3.7E-08	cell wall organization or biogenesis
26	4	32	1.420	7.9E-05	carbohydrate biosynthetic process
27	8	128	1.282	1.1E-06	intracellular signaling pathway
28	8	136	1.251	1.8E-06	signaling pathway
29	8	136	1.251	1.8E-06	signaling
30	5	70	1.184	1.3E-04	amine metabolic process
31	6	106	1.115	7.9E-05	site of polarized growth

Results obtained from the FuncAssociate 2.0 web application.

[a]N - the number of congruent genes that have the GO attribute.

[b]X - total number of interacting genes covered by our array that have the GO attribute.

[c]LOD - Logarithm (base 10) of the odds ratio.
