# Supplementary material for: A Simple Yeast-Based Strategy to Identify Host Cellular Processes Targeted by Bacterial Effector Proteins
Source: PLoS One. 2011 Nov 15;6(11):e27698. doi: 10.1371/journal.pone.0027698 (PMC3216995; doi:10.1371/journal.pone.0027698)
Supplement: Table S4 — GO attributes enriched among the genes congruent to XopE2 (including the congruent genes). (PDF) [file pone.0027698.s010.pdf]

Table S4. GO attributes enriched among the genes congruent to XopE2 (including the congruent genes)

Rank	GO attribute	*gas1*	*bni1*	*smi1*	*bem2*	*bck1*	*rvs167*	*spa2*	*skt5*	*myo2*	*chs5*	*chs3*	*slt2*
1	cellular cell wall organization	+	+	+	+	+	+	+	+	+	+	+	+
2	external encapsulating structure organization	+	+	+	+	+	+	+	+	+	+	+	+
3	cell wall organization	+	+	+	+	+	+	+	+	+	+	+	+
4	cellular cell wall organization or biogenesis	+	+	+	+	+	+	+	+	+	+	+	+
5	cell wall organization or biogenesis	+	+	+	+	+	+	+	+	+	+	+	+
6	site of polarized growth		+	+	+	+	+	+	+	+	+	+	+
7	cell wall chitin metabolic process								+		+	+	
8	incipient cellular bud site		+	+	+			+	+	+		+	
9	mating projection tip		+	+	+	+	+	+		+	+	+	
10	cell projection part		+	+	+	+	+	+		+	+	+	
11	cell wall polysaccharide metabolic process								+		+	+	
12	ER-nucleus signaling pathway	+		+		+		+	+		+	+	+
13	aminoglycan metabolic process								+		+	+	
14	chitin metabolic process								+		+	+	
15	cell wall macromolecule metabolic process								+		+	+	
16	cellular bud tip		+		+			+		+			+
17	cellular bud neck		+	+	+			+	+	+		+	
18	intracellular signaling pathway	+		+	+	+		+	+		+	+	+
19	signaling pathway	+		+	+	+		+	+		+	+	+
20	signaling	+		+	+	+		+	+		+	+	+
21	cellular component organization	+	+	+	+	+	+	+	+	+	+	+	+
22	sexual reproduction		+		+			+	+	+	+		+
23	actin filament-based process		+		+		+	+		+			
24	reproduction		+		+			+	+	+	+		+

A plus sign marks an association between a GO attribute and a congruent gene.
